# Ethanol production from *N*-acetyl-d-glucosamine by *Scheffersomyces stipitis* strains

**DOI:** 10.1186/s13568-016-0267-z

**Published:** 2016-10-03

**Authors:** Kentaro Inokuma, Tomohisa Hasunuma, Akihiko Kondo

**Affiliations:** 1 Graduate School of Science, Technology and Innovation, Kobe University, 1-1 Rokkodai, Nada, Kobe, 657-8501 Japan; 2 Biomass Engineering Program, RIKEN, 1-7-22 Suehiro-cho, Tsurumi-ku, Yokohama, Kanagawa 230-0045 Japan

**Keywords:** Ethanol, *N*-acetylglucosamine, Chitin, Bioconversion, *Scheffersomyces stipitis*, Non-conventional yeast

## Abstract

*N*-acetyl-d-glucosamine (GlcNAc) is the building block of chitin, which is one of the most abundant renewable resources in nature after cellulose. Therefore, a microorganism that can utilize GlcNAc is necessary for chitin-based biorefinery. In this study, we report on the screening and characterization of yeast strains for bioethanol production from GlcNAc. We demonstrate that *Scheffersomyces* (*Pichia*) *stipitis* strains can use GlcNAc as the sole carbon source and produce ethanol. *S. stipitis* NBRC1687, 10007, and 10063 strains consumed most of the 50 g/L GlcNAc provided, and produced 14.5 ± 0.6, 15.0 ± 0.3, and 16.4 ± 0.3 g/L of ethanol after anaerobic fermentation at 30 °C for 96 h. The ethanol yields of these strains were approximately 81, 75, and 82 % (mol ethanol/mol GlcNAc consumed), respectively. Moreover, *S. stipitis* NBRC10063 maintained high GlcNAc-utilizing capacity at 35 °C, and produced 12.6 ± 0.7 g/L of ethanol after 96 h. This strain also achieved the highest ethanol titer (23.3 ± 1.0 g/L) from 100 g/L GlcNAc. To our knowledge, this is the first report on ethanol production via fermentation of GlcNAc by naturally occurring yeast strains.

## Introduction

The search for practical petroleum substitutes from renewable resources has become a global priority to combat the rapid rise in atmospheric carbon dioxide levels. Chitin, which is the principal structural component of cell walls of fungi, yeasts, and algae, exoskeletons of insects, shells of crustaceans, and microfilarial sheathes of nematodes (Flach et al. 1992), is one of the most abundant renewable resources in nature following cellulose (Howard et al. 2003). The estimated annual production of chitin on the Earth is on the order of 10^10^ to 10^11^ tons (Gooday 1990). Chitin is currently extracted from crab and shrimp shell wastes. In shrimp production, the shells of these animals make up as much as 75 % of the waste with roughly half being chitin (Bhattacharya et al. 2007). At present, however, only a limited fraction of shell waste is being utilized for animal feed or for the isolation of chitin to be used in medicines, cosmetics, and agriculture. Moreover, the processing of shellfish leads to environmental pollution (Synowiecki and Al-Khateeb 2003). Therefore, chitin derived from unused chitinous wastes is attracting attention as an abundant substrate for potential applications in biorefinery (Hayes et al. 2008).

Chitin is a polymer of β-(1-4) linked aminosugar *N*-acetyl-d-glucosamine (GlcNAc) residues, and can be hydrolyzed by mineral acids or enzymes into GlcNAc (Cosio et al. 1982). Therefore, a microorganism that can utilize GlcNAc is necessary to establish chitin-based biorefinery. It has been reported that some native microorganisms such as *Escherichia coli* (Alvarez-Anorve et al. 2005), *Clostridium paraputrificum* (Evvyernie et al. 2001), dimorphic pathogenic fungi (Kumar et al. 2000; Inokuma et al. 2013), and some oleaginous microorganisms (Rodriguez and Dominguez 1984; Ruiz-Herrera and Sentandreu 2002; Zhang et al. 2011) can use GlcNAc for their growth and as a source of energy.

Recently, ethanol production from GlcNAc by dimorphic fungi *Mucor* species was reported (Inokuma et al. 2013). *Mucor circinelloides* NBRC6746 and *Mucor ambiguous* NBRC8092 produced approximately 18.6 and 16.9 g/L ethanol from 50 g/L GlcNAc, respectively (Inokuma et al. 2013). On the other hand, to our knowledge, the GlcNAc-utilization capacity and ethanol productivity of yeasts, which are the most commonly used microorganisms for industrial ethanol production, have not been quantitatively evaluated.

The objective of this study was to determine the feasibility of using native yeasts to convert GlcNAc into ethanol. First, a screening test was conducted among native ethanol-producing yeasts to evaluate their GlcNAc-utilization capacity. We revealed that a native *Scheffersomyces stipitis* (formerly known as *Pichia stipitis*) strain could consume GlcNAc as the sole carbon source. Second, growth assays of five native *S. stipitis* strains were performed in GlcNAc medium. Finally, anaerobic ethanol fermentation from 50 and 100 g/L of GlcNAc was performed using the *S. stipitis* strains. To our knowledge, this is the first report of ethanol production via fermentation of GlcNAc by naturally occurring yeast strains.

## Materials and methods

### Strains and media

The yeast strains used in this study are listed in Table 1. *Kluyveromyces lactis* NRRL Y-1140 was obtained from the US Department of Agriculture-Agricultural Research Service (USDA-ARS) Culture Collection, and other strains were obtained from the NITE Biological Resource Center (NBRC). Yeast cells were pre-cultured in 5 mL of yeast extract peptone dextrose (YPD) medium [10 g/L yeast extract, 20 g/L Bacto-peptone (Difco Laboratories, Detroit, MI, USA), and 20 g/L glucose] in a shaker incubator (180 rpm at 25 °C for *S. stipitis* NBRC1720 and 10006 and 30 °C for other strains; BR-43FL; Taitec, Saitama, Japan) for 18 h. The yeast cells were harvested by centrifugation at 1000×*g* for 5 min, and then washed twice with distilled water. The washed cells were used for screening, growth assays, and ethanol fermentation as described below. Synthetic GlcNAc (SGN) medium [6.7 g/L yeast nitrogen base without amino acids (Difco Laboratories) and 50 g/L GlcNAc (Wako Pure Chemicals, Osaka, Japan)] was used to screen GlcNAc-utilizing strains. YPGN50 medium containing 10 g/L yeast extract, 20 g/L Bacto-peptone, and 50 g/L GlcNAc and YPGN100 medium containing 10 g/L yeast extract, 20 g/L Bacto-peptone, and 100 g/L GlcNAc were used for ethanol fermentation and growth assays.

**Table 1:** Yeast strains used in this study

Yeast strains	Other culture collection no.	Source
*Saccharomyces cerevisiae* S288c	NBRC1136	NBRC
*Kluyveromyces marxianus* NBRC1777		NBRC
*Kluyveromyces lactis* NRRL Y-1140	CBS2359, NBRC1267	USDA-ARS
*Pichia pastoris* NBRC1013		NBRC
*Scheffersomyces stipitis* NBRC1687	CBS5773, NRRL Y-7124	NBRC
*Scheffersomyces stipitis* NBRC1720	CBS7124	NBRC
*Scheffersomyces stipitis* NBRC10006	CBS7125	NBRC
*Scheffersomyces stipitis* NBRC10007	CBS7126, NRRL Y-17104	NBRC
*Scheffersomyces stipitis* NBRC10063	CBS6054, NRRL Y-11545	NBRC

### Screening of GlcNAc-utilizing strains

After pre-cultivation and washing, yeast cells were inoculated in 5 mL SGN medium in test tubes to an initial OD_600_ of 0.1, and then cultivated at 30 °C in a shaker incubator (180 rpm; BR-43FL; Taitec). After cultivation for 24 h, the GlcNAc consumption of each strain was determined using high performance liquid chromatography (HPLC) as described below.

### Growth assay

Growth assays of yeast strains were performed in L-shaped test tubes by using a TVS062CA Bio-photorecorder (Advantec Toyo, Tokyo, Japan). Pre-cultivated cells were inoculated in 5 mL of YPGN50 medium to an initial OD_600_ of 0.1. The yeast cells were cultured microaerobically (70 rpm), and OD_600_ of the cell suspension was automatically measured every 30 min. The final cell density (OD_600_) after 72 h of cultivation was measured by a UV–VIS spectrophotometer (UVmini-1240, Shimadzu, Kyoto, Japan). The μmax values were calculated as described previously (Inokuma et al. 2015).

### Ethanol fermentation of GlcNAc

Ethanol fermentation of GlcNAc was anaerobically performed in closed 100 mL bottles equipped with a CO_2_ outlet. Yeast cells were inoculated in 20 mL of YPGN50 or 100 medium at an initial OD_600_ of 0.1. Fermentation was initiated by the addition of yeast cells into the fermentation medium, followed by rotation in a shaker incubator (180 rpm; BR-43FL; Taitec). The culture broth was sampled every 24 h, and its GlcNAc, ethanol, and acetate concentrations were determined using HPLC as described below.

### Analytical methods

The concentrations of GlcNAc, ethanol, and acetate in the culture medium were determined using HPLC (Shimadzu). An Aminex HPX-87H column (Bio-Rad, Hercules, CA, USA) was used together with a Bio-Rad 125–0131 guard cartridge (Bio-Rad) and a refractive index detector (RID-10A, Shimadzu). The HPLC system was operated at 65 °C with 5 mM H_2_SO_4_ (flow rate, 0.6 mL/min) as the mobile phase.

## Results

### Screening of GlcNAc-utilizing strains

**Fig. 1:**
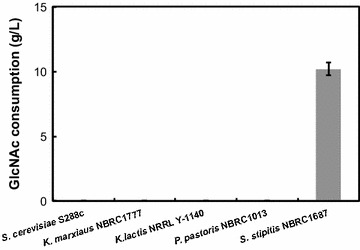
GlcNAc-utilizing capacity of native ethanol-producing yeasts in synthetic GlcNAc medium. *Error bars* indicate standard deviations of three independent experiments

To screen for yeast strains that could utilize GlcNAc, native ethanol-producing yeasts were cultivated aerobically at 30 °C in the synthetic medium containing 50 g/L of GlcNAc as the sole carbon source (SGN medium). After 24-h cultivation, the GlcNAc consumption of these strains was evaluated using HPLC as described in the Materials and Methods. The results are shown in Fig. 1. Among the tested strains, only *S. stipitis* NBRC1687, which is the type strain of *S. stipitis,* could consume GlcNAc as the sole carbon source, and no significant GlcNAc consumption (<1.0 g/L) and cell growth were observed in the other strains. The NBRC1687 strain consumed 10.2 ± 0.5 g/L of GlcNAc and the OD_600_ reached 12.8 ± 0.4 after 24-h cultivation.

### Growth assay of *S. stipitis* strains in GlcNAc medium

Microaerobic cultivation of five *S. stipitis* strains obtained from NBRC (Table 1) was performed in YPGN50 medium. The yeast cells were cultured at different temperatures (23–37 °C for NBRC1720 and 10006 strains, and 25–40 °C for NBRC1687, 10007 and 10063 strains) for 72 h, and the growth rates and final cell densities of these strains were evaluated. The growth profiles of these strains are shown in Table 2. Significant cell growth was observed in all *S. stipitis* strains tested in this study. NBRC1720 and 10006 strains exhibited cell growth in the range of 23–30 °C, while no significant growth was observed above 35 °C. On the other hand, NBRC1687, 10007, and 10063 strains were able to withstand cultivation temperatures in the range of 25–37 °C, and increasing growth temperature up to 35 °C did not significantly affect the growth rates. The highest μmax values of these strains were observed at 25 °C (NBRC1720 and 10006) and 30 °C (NBRC1687, 10007, and 10063) (Table 2). Consequently, the next ethanol fermentation experiments were carried out at 25 °C (NBRC1720 and 10006) and 30 °C (NBRC1687, 10007, and 10063).

**Table 2:** Growth profiles of *S. stipitis* strains in YPGN50 medium under aerobic conditions

*S. stipitis* strains	Temperatures (°C)	Lag time (h)	μmax (/h)	Final cell density (OD_600_ at 72 h)
NBRC1687	25	8.0	0.308 ± 0.012	11.3 ± 0.2
	30	6.0	0.407 ± 0.012	11.4 ± 0.1
	35	6.0	0.353 ± 0.018	11.9 ± 0.1
	37	10.0	0.311 ± 0.033	11.6 ± 0.2
	40	25.0	0.390 ± 0.128	2.7 ± 0.3
NBRC1720	23	13.0	0.314 ± 0.029	9.5 ± 0.3
	25	12.5	0.317 ± 0.018	9.6 ± 0.1
	30	16.5	0.209 ± 0.023	9.0 ± 0.8
	35	–	–	<0.1
	37	–	–	<0.1
NBRC10006	23	13.5	0.239 ± 0.005	9.8 ± 0.3
	25	12.0	0.246 ± 0.004	9.7 ± 0.1
	30	15.5	0.135 ± 0.033	6.5 ± 2.5
	35	–	–	<0.1
	37	–	–	<0.1
NBRC10007	25	8.0	0.299 ± 0.004	13.6 ± 0.3
	30	6.0	0.403 ± 0.012	17.4 ± 0.6
	35	6.0	0.369 ± 0.005	17.1 ± 0.1
	37	9.0	0.215 ± 0.032	12.2 ± 2.7
	40	–	–	<0.1
NBRC10063	25	7.0	0.333 ± 0.007	13.4 ± 0.1
	30	5.0	0.418 ± 0.007	12.1 ± 0.3
	35	6.0	0.405 ± 0.015	14.6 ± 0.4
	37	7.0	0.341 ± 0.017	13.7 ± 0.9
	40	–	–	<0.1

The averages for three independent experiments are shown with their standard deviations

### Ethanol fermentation from 50 g/L of GlcNAc

**Fig. 2:**
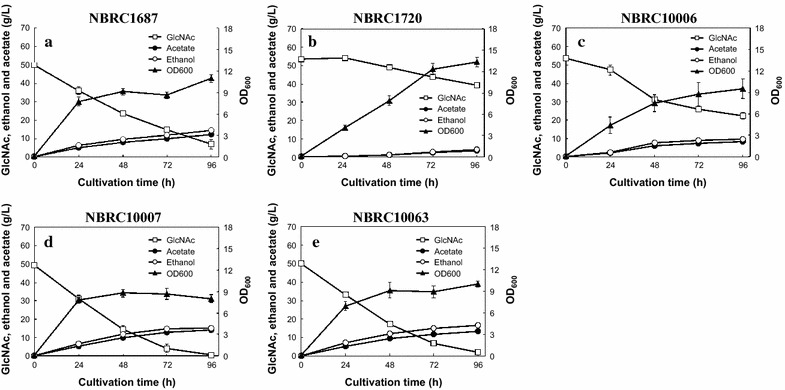
Time-course of anaerobic ethanol fermentation from 50 g/L GlcNAc by *S. stipitis* NBRC1687 (**a**), 1720 (**b**), 10006 (**c**), 10007 (**d**), and 10063 (**e**) strains at 30 °C. *Error bars* indicate standard deviations of three independent experiments

Ethanol fermentation from 50 g/L of GlcNAc using *S. stipitis* strains was performed under anaerobic conditions because *S. stipitis* has the characteristics of a Crabtree-negative yeast and is unable to produce ethanol in the presence of fermentable sugars under aerobic conditions (Jeffries and Shi 1999; Passoth et al. 1996). The results are shown in Fig. 2. All *S. stipitis* strains tested in this study could produce ethanol from GlcNAc, while their ethanol productivities varied depending on the strains. Among these strains, NBRC1687, 10007, and 10063 consumed most of the supplied 50 g/L GlcNAc. These strains produced 14.5 ± 0.6, 15.0 ± 0.3, and 16.4 ± 0.3 g/L of ethanol after 96-h fermentation, and their ethanol yields were approximately 0.338, 0.306, and 0.342 (g/g GlcNAc consumed), respectively (Fig. 2a, d, e). Since the theoretical maximum ethanol yield from GlcNAc by the GlcNAc catabolic pathway (Biswas et al. 2007) and the glycolysis pathway via fructose-6-phosphate is 2 mol ethanol/mol GlcNAc consumed (0.417 g ethanol/g GlcNAc consumed), the ethanol yields of these strains represent approximately 81, 75, and 82 % of the theoretical yield, respectively.

**Fig. 3:**
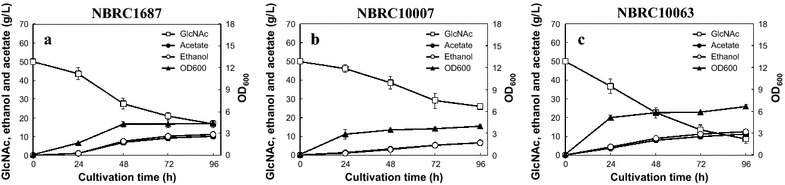
Time-course of anaerobic ethanol fermentation from 50 g/L GlcNAc by *S. stipitis* NBRC1687 (**a**), 10007 (**b**), and 10063 (**c**) strains at 35 °C. *Error bars* indicate standard deviations of three independent experiments

To evaluate the fermentation ability of these strains at elevated temperature, we also performed ethanol fermentation at 35 °C using NBRC1687, 10007, and 10063 strains (Fig. 3). Although the GlcNAc consumptions, cell growth, and the ethanol titers of these strains were lower than those at 30 °C, NBRC10063 demonstrated relatively high fermentation ability at 35 °C. This strain produced 12.6 ± 0.7 g/L of ethanol from GlcNAc, and the yield was approximately 0.303 g/g after 96 h (Fig. 3c).

### Ethanol fermentation from 100 g/L of GlcNAc

**Fig. 4:**
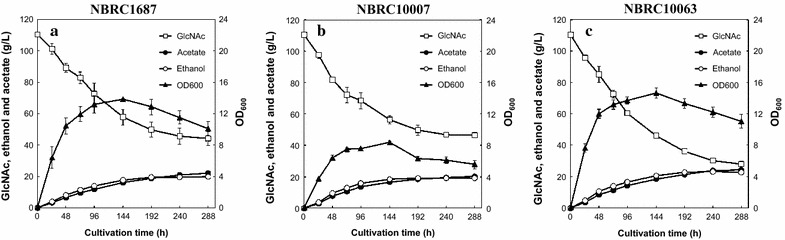
Time-course of anaerobic ethanol fermentation from 100 g/L GlcNAc by *S. stipitis* NBRC1687 (**a**), 10007 (**b**), and 10063 (**c**) strains at 30 °C. *Error bars* indicate standard deviations of three independent experiments

Ethanol fermentation from 100 g/L of GlcNAc was performed at 30 °C to investigate the maximum ethanol titer of the *S. stipitis* strains. The results are shown in Fig. 4. These strains could use more than 50 g/L of GlcNAc, while their GlcNAc consumption and ethanol production rates gradually decreased over time and no significant ethanol production was observed after 240 h. Among these strains, NBRC10063 showed the highest GlcNAc consumption (80.2 ± 1.2 g/L) and ethanol titer (23.3 ± 1.0 g/L) after 240 h cultivation. In all fermentation experiments shown in Fig. 4, initial pH was 6.6 and the final pH settled at 5.8–5.9 after 288 h.

## Discussion

In this study, we performed screening and characterization of yeast strains for bioethanol production from GlcNAc. We found that natural *S. stipitis* strains can use GlcNAc as the carbon source and produce ethanol. *S. stipitis* is able to ferment a wide range of sugars including galactose, mannose, xylose, and cellobiose along with mannan and xylan oligomers (Du Preez et al. 1986; Jeffries and Van Vleet 2009), and is widely studied for its capacity to ferment D-xylose to ethanol. However, there have been no studies concerning its capacity to ferment GlcNAc to ethanol. To our knowledge, this is the first report of ethanol production from GlcNAc by naturally occurring yeast strains.

*Scheffersomyces stipitis* NBRC10063 gave the highest ethanol titer and yield among the *S. stipitis* strains used in this study (Fig. 2). In anaerobic fermentation at 30 °C, this strain produced 16.4 g/L ethanol from 50 g/L GlcNAc. This ethanol titer is similar to that reported for *M. circinelloides* (18.6 g/L) and *M. ambiguus* (16.9 g/L) (Inokuma et al. 2013). Moreover, this strain achieved the highest GlcNAc consumption (80.2 g/L) and ethanol titer (23.3 g/L) from 100 g/L GlcNAc after 240 h cultivation. Although Wendland et al. (2009) reported ethanol production from GlcNAc by recombinant *S. cerevisiae* strains transduced with four genes required for the GlcNAc catabolic pathway from *Candida albicans*, the ethanol titers of these recombinant yeasts (around 3 g/L after 11 days) were much lower than that of NBRC10063. NBRC10063 also showed relatively high ethanol production from GlcNAc at 35 °C (Fig. 3). Applicability to high fermentation temperatures is important for efficient simultaneous saccharification and fermentation (SSF) processes, because high-temperature fermentation will reduce the cooling cost and risk of contamination and enable stable fermentation even in tropical countries (Banat et al. 1998).

In ethanol fermentation from 100 g/L of GlcNAc, GlcNAc consumption and ethanol production rates of *S. stipitis* strains gradually decreased over time and no significant ethanol production was observed after 240 h even though GlcNAc still remained (Fig. 4). One possible cause for the reduction of the fermentation efficiency is acetate accumulation. Acetate is an inhibitor of ethanol fermentation by yeast. Vanzyl et al. (1991) reported that the volumetric rate of ethanol production of *S. stipitis* CBS7126 (NBRC10007) from xylose was inhibited 50 % by acetate at concentration of 13.8 g/L at pH 6.5 under anaerobic condition. In this study, *S. stipitis* strains produced more than 20 g/L acetate as a byproduct after 288 h cultivation (Fig. 4). Similar acetate accumulation has been observed in ethanol fermentation from GlcNAc using *Mucor* species (Inokuma et al. 2013). Singh and Datta (1979) have reported the GlcNAc-catabolic pathway in *C. albicans* as follows. GlcNAc transported across the cell membrane can be phosphorylated to form GlcNAc 6-phosphate (GlcNAc 6P) by kinase. Then GlcNAc 6P is deacetylated to glucosamine 6-phosphate (GlcN 6P) followed by deamination to produce fructose 6-phosphate by GlcN 6P deaminase. Therefore, it can be inferred that most of the accumulated acetate is generated by the deacetylation of GlcNAc 6P to GlcN 6P. For further improvement of ethanol production from GlcNAc using *S. stipitis* strains, removal or convert of the accumulated acetate is necessary. Recently, several reports have demonstrated improvement of ethanol yields in *S. cerevisiae* by anaerobic reduction of acetate to ethanol (Henningsen et al. 2015). A similar approach would be applicable for the improvement of the ethanol yield from GlcNAc by *S. stipitis*. If *S. stipitis* strains can convert acetate into ethanol, these strains will be able to produce a maximum 3 mol of ethanol from 1 mol of GlcNAc (0.625 g ethanol/g GlcNAc) theoretically. The theoretical yield is much higher than those from glucose (0.514 g/g) and xylose (0.511 g/g).

Compared with *S. cerevisiae,* which is widely used in industrial ethanol fermentation, heterologous gene expressions and targeted gene deletions of *S. stipitis* are more difficult due to its alternative codon system and frequent random (nonhomologous) integration (Jeffries and Van Vleet 2009). However, researchers have been developing genetic transformation systems based on auxotrophic markers (Yang et al. 1994; Lu et al. 1998; Piontek et al. 1998) and drug resistance markers (Laplaza et al. 2006), the loxP/Cre excision system (Laplaza et al. 2006), and expression vectors available for *S. stipitis* (Den Haan and Van Zyl 2001; Klabunde et al. 2003). A summary of strain development and genetic tools useful for *S. stipitis* has been published (Jeffries 2008). Furthermore, Jeffries et al. (2007) sequenced the genome of *S. stipitis* NBRC10063 (CBS6054, NRRL Y-11545) strain. These genetic tools and genome information can aid in the genetic engineering of *S. stipitis* strains such as the conversion of acetate into ethanol and the expression of heterologous chitinase genes.

In this study, we evaluated yeast strains based on their cell growth and ethanol production in GlcNAc medium. On the other hand, it has been reported that enzymatic chitin degradation is significantly enhanced by hydrochloric acid treatment (Inokuma et al. 2013). Therefore, comparison of tolerance of *S. stipitis* strains to hydrochloric acid and other compounds present in the acid-treated chitin hydrolysate would be necessary for further evaluation of their availabilities. If strains are both tolerant to these compounds and can utilize GlcNAc well, they will be more promising for utilization of chitinous wastes.

In this study, we demonstrated that native *S. stipitis* strains could use GlcNAc as the sole carbon source and produce ethanol efficiently. *S. stipitis* NBRC10063 showed the highest growth rate, GlcNAc-utilizing capacity, ethanol productivity, and thermal stability in YPGN medium among the yeast strains tested in this study. Our results suggest that the NBRC10063 strain should be regarded as a promising candidate for use in bioethanol production from chitinous waste in the future. However, further analysis of the GlcNAc metabolic pathway of this yeast is necessary to identify the reasons for its high GlcNAc-utilization capacity.

## Abbreviations

GlcNAc: *N*-acetyl-d-glucosamine
GlcNAc 6P: GlcNAc 6-phosphate
GlcN 6P: glucosamine 6-phosphate
USDA-ARS: US Department of Agriculture-Agricultural Research Service
NBRC: NITE Biological Resource Center
YPD: yeast extract peptone dextrose
SGN: synthetic GlcNAc
YPGN50: yeast extract peptone with 50 g/L GlcNAc
YPGN100: yeast extract peptone with 100 g/L GlcNAc
SSF: simultaneous saccharification and fermentation
